# Safety Assessment of Genetically Modified Feed: Is There Any Difference From Food?

**DOI:** 10.3389/fpls.2019.01592

**Published:** 2019-12-11

**Authors:** Paula A. Giraldo, Hiroshi Shinozuka, German C. Spangenberg, Noel O.I. Cogan, Kevin F. Smith

**Affiliations:** ^1^Faculty of Veterinary and Agricultural Sciences, The University of Melbourne, Parkville, Melbourne, VIC, Australia; ^2^Agriculture Victoria Research, AgriBio, The Centre for AgriBiosciences, Melbourne, VIC, Australia; ^3^School of Applied Systems Biology, La Trobe University, AgriBio, The Centre for AgriBiosciences, Melbourne, VIC, Australia; ^4^Agriculture Victoria Research, Hamilton, VIC, Australia

**Keywords:** traceability, environment, toxicological, allergenicity, forage, transgenic crops, regulatory framework, genome-editing

## Abstract

Food security is one of major concerns for the growing global population. Modern agricultural biotechnologies, such as genetic modification, are a possible solution through enabling an increase of production, more efficient use of natural resources, and reduced environmental impacts. However, new crop varieties with altered genetic materials may be subjected to safety assessments to fulfil the regulatory requirements, prior to marketing. The aim of the assessment is to evaluate the impact of products from the new crop variety on human, animal, and the environmental health. Although, many studies on the risk assessment of genetically modified (GM) food have been published, little consideration to GM feedstuff has been given, despite that between 70 to 90% of all GM crops and their biomass are used as animal feed. In addition, in some GM plants such as forages that are only used for animal feeds, the assessment of the genetic modification may be of relevance only to livestock feeding. In this article, the regulatory framework of GM crops intended for animal feed is reviewed using the available information on GM food as the baseline. Although, the majority of techniques used for the safety assessment of GM food can be used in GM feed, many plant parts used for livestock feeding are inedible to humans. Therefore, the concentration of novel proteins in different plant tissues and level of exposure to GM feedstuff in the diet of target animals should be considered. A further development of specific methodologies for the assessment of GM crops intended for animal consumption is required, in order to provide a more accurate and standardized assessment to the GM feed safety.

## Introduction

The growth of the human population will create significant challenges for agricultural production, making food security a growing issue. Nearly 870 million people suffer from malnutrition, most of them in the developing countries of Africa, Asia, and South America (World Hunger, 2013). Additionally, climate change and environmental degradation are currently reducing the available agricultural land, creating additional challenges to fill the increasing food demand (Hanjra and Qureshi, 2010). The use of modern biotechnology, including genetic modification techniques, has been proposed as a way to reduce the environmental footprint, by improving food quality and increasing productivity (Barros et al., 2019).

Animal derived food is a major contributor to human nutrition and health, largely through supplying protein. It also plays a crucial role in rural economies of most developing countries, particularly in dry areas (Godfray et al., 2010). In order to produce enough protein for the growing global population, farming systems are increasing the pressure on land, water resource use and biodiversity conservation. Solutions to face climate change and high demand for natural resources are urgently needed, especially in the area of forage production (Sakadevan and Nguyen, 2017), since it compromises nearly 80% of the world agricultural land and provides the grazing feed-base for the dairy and red meat industries (Bruinsma, 2017).

Implementation of molecular breeding technologies in forage crops can enhance the agricultural sector through increases in productivity, more efficient use of natural resources and decreases in environmental impacts (Ramessar et al., 2010). Breeding programs target the development of genetic solutions for forage quality limitations, pest and disease resistance, nutrient acquisition efficiency, tolerance to abiotic stresses, and the targeted modification of growth and development (Smith et al., 2007).

Transgenesis is a classical DNA modification methodology, which enables production of crops with desired traits based on the introduced transgene(s). Genome editing technologies were more recently established, which enable alteration of the DNA to produce defined multination(s) and/or insertion of foreign gene(s) at the targeted site(s), in contrast to GM where the insertion is random (Sprink et al., 2016). Genetic modification is typically defined as alteration of the genetic material of an organism in a way, which does not naturally occur (Wolt et al., 2015). Crop cultivars developed through use of transgenesis, therefore, have been regard as GM organisms (Hundleby and Harwood, 2019).

Transgenic crops can be further divided into four classifications/classes, according to the structure and strategy for transgenesis (Lin and Pan, 2016). Most GM commercially available today are categorized as the first class of transgenics, also called single trait transgenics. These crops typically contain common transgenic elements, such as the 35s promoter sequence of cauliflower mosaic virus, and nopaline synthase terminator (nos-T) from *Agrobacterium* (Shaw et al., 1984; Wehrmann et al., 1996). The second class transgenics have stacked modified traits, and these varieties usually result from hybrid crosses of first-generation GM crop varieties. The hybrid cross procedure may increase economic values of the GM variety with a lower development cost. The third class of transgenics are so-called near-intragenics, these are GM crops where the transgene construction originates from the host with some minimal modifications. The last class are more related to true intragenic or cisgenic technologies, where the transgene is comprised of only products and elements from the host, without modifications and the only difference with its conventional counterpart is the specific order and insertion loci of the transgenes (Rommens et al., 2007; Jacobsen and Schouten, 2009; Lin and Pan, 2016).

On the other hand, genome editing technologies use biological tools such as sequence-specific nucleases to generate desired modifications within plant genomes, allowing the introduction of one or more transgenes at a specific locus, the removal of unwanted DNA from the host, or the control in the expression of endogenous or synthetic genes (Songstad et al., 2017).

Since approvals for commercialization of early-generation transgenic crops, safety issues for human consumption have been mainly considered. GM crops as animal feed and GM forage have not been considered as the primary target of the regulatory framework. Although there are some animal feedstuffs that crossover with human consumption (e.g., grain), there are many plants and plant parts that are not directly consumed by humans but are exclusively used by livestock as feed. This review article aims to summarize and discuss the elements needed for the safety assessment of GM crops for animal feed purposes, using the available information on the current practice of safety assessment that the product would be subjected to, as the baseline.

## GM Feed

The market share of GM products has rapidly increased from commercialization of the early generation of GM crops in the 1990s, (I.S.A.A.A., 2017). The major GM crops available in the market are soybean (*Glycine max* L. Merr.) with 77% of the global area for individual crops (94.1 million hectares), maize (*Zea mays* L.) at 32% (59.7 million hectares), cotton (*Gossypium arboretum* L.) at 80% (24.21 million hectares), and canola (*Brassica napus* L.) 30% (10.2 million hectares). The commercial use of transgenesis-delivered crops cultivars has also recently expanded to more species including sugar beet (*Beta vulgaris* L.), papaya (*Carica papaya* L.), squash (*Cucurbita* L.), eggplant *Solanum melongena* L.), potatoes (*Solanum tuberosum* L.), and apples (*Malus pumila* Miller, 1768), and these products are already commercially available in US (I.S.A.A.A., 2017). A meta-analysis including 147 food and feed crops, also has revealed that the adoption of GM technology has decreased the use of chemical pesticides by 37%, increased crop yields by 22%, and increased farmer profits by 68% (Klümper and Qaim, 2014).

GM crops can be traded as food and feed products (Panel on Genetically Modified Organisms, 2010). The products are classified as GM food, when the direct consumers are mainly humans, and the products only intended for animal consumption are regarded as GM feed. However, a range of GM crops, such as maize, soybean, and canola, are used as both food and feed (**Figure S1**). Most GM crops available in the market, except for alfalfa (*Medicago sativa L*. ssp. sativa) and creeping bentgrass (*Agrostis stolonifera*) have been assessed as GM food, because they can be consumed by humans. On the basis of biomass, between 70 to 90% of all GM crops, however, are estimated to be used in farm as animal feed (Flachowsky et al., 2012).

In contrast to GM food crops, only a few types of GM forage products have been commercially released. Alfalfa is an economically important legume forage mainly in temperate regions. The first GM forage crop commercialized in US was the Roundup Ready^®^ Alfalfa from Forage Genetics International (Nampa, ID, US), which can be categorized as a first generation transgenic (van Deynze et al., 2004).The herbicide-resistance trait was produced through inserting two copies of an *Agrobacterium*-derived gene (*cp4 epsps*) of which translational product (EPSPS; 5-enolpyruvylshikimate-3-phosphate synthase) contribute to glyphosate-tolerance. Such insertion of the foreign gene allows post-emergence applications of glyphosate-based herbicides for weed control (Putnam et al., 2016).

Following to Roundup Ready^®^ Alfalfa, another first-generation transgenic, HarvXtra™ Alfalfa (Forage Genetics International) was developed and commercially released. Using RNA interference (RNAi)-based gene suppression mechanics, the lignin content and composition are modified in this cultivar. A transgene cassette including an inverted repeat of the interference-targeting sequence was introduced using *A. tumefaciens* for suppression of caffeoyl-CoA 3-O-methyltransferase (CCoAOMT), which is involved in lignin biosynthesis. The inverted repeat was designed to be transcribed under control of the phenylalanine ammonia-lyase (PAL)2 promoter from common bean (*Phaseolus vulgaris* L.) for vascular tissue-specific expression, allowing the desired suppression of lignin biosynthesis without negative effects on forage yield (Barros et al., 2019).

Perennial ryegrass (*Lolium perenne* L.) is also one of most important forage species in the temperate regions. Although several transgenic perennial ryegrass plants with potentially economically useful traits have been developed, none of them has been commercially released. Using a similar approach to HarvXtra™ Alfalfa, low-lignin perennial ryegrass individuals have been generated (Hu et al., 2013). In the transgenic perennial ryegrass, lignin biosynthesis-related genes were down-regulated based on the RNAi mechanisms. As the transgenic low-lignin plants may have increased digestibility for cattle, the lignin biosynthesis-controlling technology may be useful for production of optimal feedstocks. The Chimeric REpressor Gene-Silencing Technology (CRES-T) is a newly developed transgenesis-based approach for functional analysis of transcription factors in plants (Mitsuda et al., 2011). A CRES-T transgenic construct was developed to target a zinc finger transcription factor gene, which is negatively related to salt tolerance (Cen et al., 2016). Transgenic perennial ryegrass plants with the CRES-T construct showed a higher tolerance to salt stress (up to 300 mM NaCl). Interestingly, the transgenic perennial ryegrass also showed a vigorous phenotype under non-salt stress condition, which may be useful for forage breeding purposes.

Perennial ryegrass plants with a high-energy trait have been also developed, using the transgenic technologies. A synthesized construct of perennial ryegrass 6-glucose fructosyltransferase (6G-FFT) and sucrose:sucrose 1-fructosyl-transferase (1SST) genes were introduced into the perennial ryegrass genome, aiming enhancement of fructan biosynthesis in the leaf blades (Panter et al., 2017). The transgenic plants showed a substantial increment in fructan accumulation in leaf blades, as well as enhanced biomass production. These traits could be beneficial for the livestock industry, as leaf blades are the major part as feed for grazing ruminants (Lee et al., 2003). The transgenic plants were generated through insertion of manipulated perennial ryegrass genes, and this cultivar can be regarded as a third generation transgenic.

Improvement of biotic stress tolerance in white Clover (*Trifolium repens* L.), an important pasture legume in temperate regions, has been also developed through the generation of plants immune to infection by Alfalfa mosaic virus (AMV) (Smith et al., 2007). Although, this AMV resistant clover has not been commercially released yet, different studies have demonstrated the expression and stability of the viral coat protein gene encoded, by the sub-genomic RNA4 of AMV in white clover, under glass house and field conditions (Panter et al., 2012; Smith and Spangenberg, 2016).

Forage crops improvements, *via* genetic modifications and genome editing, have the potential to play a key role in fulfilling the increasing demand for animal products. Therefore, risk assessment must ensure its safety for humans, animals, and the environment, in order to have an agricultural system economically and environmentally sustainable.

## GM or GE?

Genome edited (GE) plants are gaining popularity and are still classified as GMs in some jurisdictions, so the authors considered it necessary to address the issues on these crops as well in this manuscript. Both GM and GE crops and their products are required to be subjected to rigorous evaluations as a part of several regulatory requirements before their commercial release into the market. The legislation for GM and GE crops is based on the principle of assuring the safety of humans, animals, and the environment. Comprehensive studies on the risk assessment of GM food crops have been published previously (König et al., 2004; Delaney, 2015; Domingo, 2016; Gurău and Ranchhod, 2016; Dadgarnejad et al., 2017; Tutel’yan, 2017; De Santis et al., 2018; Kumar et al., 2018).

Some variation between regulatory frameworks of GM crops exists across jurisdictions. In the US, safety assessment of new GM crops is mainly performed though comparison with conventional counterparts, to provide scientific evidence that GM products can be considered as safe as a conventional product, if the characteristics and composition are substantially equivalent (FAO/WHO, 2000). The assessment of the Canadian authority also focuses on the products itself (Alexandrova et al., 2005). European regulation focuses more on the certification of the genetic modification process, instead of the products (European Parliament, 2001), as well as Argentina, Japan, and South Africa (Seyran and Craig, 2018). The Australian regulatory authority, the Office of the Gene Technology Regulator (OGTR), has followed a safety assessment model promoted by the World Trade Organization (WTO). Such model has provided as general guideline that safety assessment of GM plants should include consideration of the risk for humans, animals, and environment, and should be science-based (Dibden et al., 2013).

Similarly, there is variation in the regulation of GE products compare with GM across jurisdictions with much of the debate being polarized (Jones, 2015). Some regulatory bodies argue that genome editing techniques are very similar to those used to produce GMOs, so they should be regulated similarly (Seyran and Craig, 2018). However, the scientific community argues that unintended effects can always occur, regardless of the types of techniques used for crop development (Fernandez and Paoletti, 2018).

From a scientific point of view, the number of alleles changed in the process of traditional breeding, such as crossing and selection of superior genotype combinations, should be typically higher than that of transgenesis or genome-editing approach. With other breeding techniques such as radiation and chemical-based mutagenesis, there is no established method for most crop species, to accurately assess the number of genes changed. In case of soybean, a combination of high-throughput DNA sequencing and molecular cytogenetics-based copy number variations assay, suggested that single nucleotide substitutions and structural variations generated during *Agrobacterium*-based transgenesis, was substantially less than those of radioactive-based mutagenesis (Anderson et al., 2016). The same approaches, also indicated that the frequencies of single nucleotide substitutions and structural variations generated through the transgenesis and mutagenesis, were considerably less than those found between existing soybean cultivars.

Different breeding techniques and their requirement for safety assessment before commercialization are described in **Figure S2**. Traditional breeding and mutagenesis, in general, change a high number of genes and mutations generally involve loss of function, while GM offers the advantage of knowing the actual gene(s) being inserted and usually involves a gain of function (Gepts, 2002). Nevertheless, the regulatory system assesses plants resulting from hybridization, radiation, or chemical induced mutagenesis, which may produce thousands of uncharacterized random mutations, as non-GM crops (Urnov et al., 2018). A recently published article that evaluates the impact of the risk assessment on public acceptance, concluded that the rigorous regulatory vigilance of modern biotechnology (transgenesis and gene editing), leads to public distrust and contribute to the idea that GM crops are unsafe. Therefore, a risk-disproportionate regulation of these technologies not only confuses the purpose of risk assessment, but also interferes with the delivery of beneficial technologies to the market (Herman et al., 2019).

As a result of such opinion polarization between the scientific community and regulatory bodies, the Court of Justice of the European Union (CJEU; Luxembourg) has decided to classify genome-editing technologies as genetic modification, submitting this plant-breeding approach to severe GM regulations and risk evaluation (Eriksson, 2019). In contrast, both the United States and Canada regulate new crop varieties according to their characteristics rather than by the method with which they are produced (Wolt, 2017). Therefore, gene-edited products without any transgene and with history of production and safe consumption, do not require special regulations (Hundleby and Harwood, 2019). The regulations implemented in the United States and Canada, is shared by a number of bodies including the UK’s Biotechnology and Biological Sciences Research Council, the German Academies, the European Plant Science Organization, and the French High Council for Biotechnology (Laaninen, 2016).

Recently, the Australian regulator gene technology regulator (OGTR) has made amendments to make the legal position of genome editing clearer. These amendments clarify that organisms modified by SDN-1 (Site-Directed Nuclease 1) techniques, present no different risk than organisms carrying naturally occurring genetic changes, and do not require unnecessary regulation (OGTR, 2019). Although in many jurisdictions, a conclusive regulatory decision has not been provided, it is possible that the position regarding GE products of the United States, Canada, Australia, and some European national regulators, will affect regulatory decisions in other countries and regions.

Both GM and GE belong to the same or similar plant biotechnology applications. In some cases, plants are transformed using recombinant DNA to introduce the GE tools, and then, they are self-pollinated or crossed to remove the incorporated DNA, leaving only the intended mutation (Metje-Sprink et al., 2020). This ability of GE to produce changes without the integration of recombinant DNA can avoid GM regulations in some countries. The reduced risk of DNA integration into the genome provided by GE, along with the indirect human exposure to GM feed, can have great implications in the commercialization of new GE feeds.

## Labeling Policies

Since the commercialization of the first GM crop in the mid-90s, some consumer groups have argued for more detailed and comprehensive labeling or extensive labeling to enable choice for the purchaser (Halle, 2008). Therefore, in response to the difficulty of maintaining zero tolerances, each jurisdiction has established a tolerance threshold for the involuntary or inevitable presence of GM material in non-GM products. If the amount of GM material in a product exceeds the tolerance threshold, the products should be labeled as containing GM material (Devos et al., 2009).

Labeling policies can include a ban on labeling, voluntary labeling indicating that a product is GM free, or mandatory labeling indicating that a product contains GM. When the latter is implemented, a legal tolerance threshold is set, and it also varies between countries, making trade and shipment of goods complex. In the US, Canada and Japan for instance, the legal tolerance threshold for conventional food and feed products has been set at 5%. Australia, New Zealand, South Africa, Brazil and China have tolerance thresholds at 1%, while in the European Union if a commercial product has more than 0.9% of GM material, it must be labeled as a GM product to inform consumers (Ramessar et al., 2010). Most countries have GM mandatory labeling laws, however, the US authority regards the nature of the product more critical than the process itself, so that GM labeling may be voluntary (Huffman and McCluskey, 2017).

To date, mandatory GM labeling laws, however, have largely excluded products from animals fed with GM feed (such as meat, milk, and eggs) as well as GM processing aids and enzymes (such as rennet for making cheese) (Van-Eenennaam and Young, 2017). In Europe, the labeling requirements (European Regulation 1830/2003; European Parliament, 2003) on GM food and feed, results in those agricultural products not requiring labeling. However, some EU member countries have established regulations and guidelines to label animal products voluntarily as non-GM, to allow consumers to choose products where no GM feed stuff were directly used in its production (Venus et al., 2018).

Even though food products derived from animals fed with GM feed crops do not require labeling, the safety assessment procedure for GM feed crops is the same than for GM food. Regulatory bodies have based such decision on the fact that it is impossible to prevent the contact of GM feed material with humans, during cultivation, transport and storage of the crops (Food Standards Australia and New Zealand, 2014).

## Safety Assessment

The scientific evidence that must be provided in the safety assessment of GM crops can vary among different legal jurisdictions (Alexandrova et al., 2005). However, a detailed molecular characterization of the transgene insertion, development of tracking and tracing methodologies to ensure legality and traceability, and environmental studies to enable coexistence frameworks, are common studies in the safety assessment of GM crops (Van Haver et al., 2003). Other studies, such as toxicological, allergenicity, nutritional, and horizontal transfer, have been performed following a case-by-case approach considering newly emerging scientific knowledge and technologies (König et al., 2004). The use of GM crops as a feed can reduce concerns around human safety and underline other features such as feeding value and nutritional equivalence (Flachowsky et al., 2002).

From an industrial point of view, the assessment required for the regulatory authorities could be divided into two groups; pre- and post-marketing issues. The former includes relatively standard technologies for all GM food and feed, such as molecular characterization and development of tracking and tracing tools for traceability purposes. Pre-marketing issues also involves technologies under development that varies on a case-by-case basis, including environmental, food/feed, toxicological, and allergenicity safety studies. Post-marketing issues are related with regulatory monitoring, which contemplate GM labeling and traceability (**Figure S3**).

### Molecular Characterization

Molecular characterization of GM crops is a full description of the structural information of the transgene and stability of the trait (Li et al., 2017). It is the foundation of all GM product safety assessments before commercialization and also serves as a baseline for the development of detection and identification tools to satisfy traceability and labeling requirements (European Parliament, 2003). Stakeholders of both GM food and feed, must provide information on the genomic locus/loci modified, copy number of the inserted transgene, insertion site, and flanking regions (Guttikonda et al., 2016).

Selection of low insertion copy number DNA transformants is preferred for the subsequent safety assessment process as it facilitates risk and hazard characterization (Tiwari and Singh, 2018). The methods most commonly used to determinate the number of transgenes integrated have been Southern blot analysis and polymerase chain reaction (PCR), in its various formats such as real-time PCR (qPCR) (Li et al., 2017).

The Southern blot analysis involves a careful selection and broad screening of restriction enzymes and designing of probes, which in some cases dependent on prior sequence information of the transgene insertion (Urquiza and Silva, 2014). However, the approach is relatively time-consuming and laborious, and also includes a manual interpretation process. In addition, the result may not accurately reflect the copy number of a transgene, if sequence rearrangements have occurred, which have affected the position(s) of the restriction enzyme recognition site(s) in the inserted transgene(s) (Yang et al., 2005).

A qPCR-based assay can more accurately quantify the copy number of transgenes by comparing to an endogenous reference sequence (endogene), which has provided a simplified alternative to Southern blot analysis (Li et al., 2017). However, identification of a single copy reference gene is occasionally difficult in crop species, due to ancestral whole genome duplications or due to polyploidy, causing complex structures and genetic redundancy (Ren et al., 2018). To overcome the identification of a reference gene and dependency on DNA calibrations, droplet digital PCR (ddPCR), a method that identifies the absolute DNA copy number in a sample, has been proposed for determination of GM copy number (Głowacka et al., 2016; Dalmira et al., 2016).

Following identification of low-copy number transformants, the precise location(s) of the transgene(s) in the crop genome is required to be identified (Park et al., 2017). DNA sequencing approaches have been used for this purpose, and this process may also identify backbone sequence(s), which were not intended to be introduced from the transformation vector into the host genome (Kononov et al., 1997). The method traditionally used for this purpose was based on Sanger sequencing (Guttikonda et al., 2016). However, the second-generation sequencing (SGS) technologies have been proposed as a new tool for molecular characterization of GM crops, due to a larger sequencing capacity and potentially higher accuracy of the resulting assembled sequence (Kovalic et al., 2012; Yang et al., 2013; Pauwels et al., 2015; Arulandhu et al., 2016).

The SGS approaches can increase speed, scalability, and automation in the selection of potential valuable events on the basis of their molecular profile, facilitating post-transformation screening (Kovalic et al., 2012; Pauwels et al., 2015; Guttikonda et al., 2016). However, these technologies do not directly provide information about the position of the insertion(s) in native DNA, due to short read lengths (50–400 base pairs) (Goodwin et al., 2016), while transgenic constructs are typically thousands of base pairs. A computational process for alignment and/or assembly of the short sequencing reads is essential for the molecular characterization purposes, and repetitive elements commonly found in plant genomes can generate problems for the alignment/assembly procedure (Liang et al., 2014).

Recently, single-molecule sequencing, also termed third-generation sequencing (TGS) platforms, have been commercialized allowing a large increase in read length up to tens of thousands of bases per read (Loose et al., 2016). Read length is limited by the input DNA fragment size, but over 300 kb have been reported (Jain et al., 2016). The increment in read lengths up to tens of thousands can facilitate a more reliable GM characterization process, by extending the sequence reads of the flanking regions present in the captured fragments and potentially solving alignment problems.

The most common TGS platforms are products from Pacific Biosciences (PacBio, Menlo Park, CA, US) and Oxford Nanopore Technologies (ONT Oxford Science Park, Oxford). PacBio uses a sequencing-by-synthesis method to capture a single DNA molecule and a circular consensus sequence (CCS) to increase accuracy. The CCS uses a circular DNA template by ligating hairpin adaptors to both ends of target double-stranded DNA, so the DNA template is sequenced multiple times to generate a continuous long read (Weirather et al., 2017). Nanopore sequencing, uses nanopores to sequence native single-stranded DNA, by measuring the changes in an electric current passed across the pore as the DNA bases pass through, disrupting the current to different levels with different nucleotides (Giordano et al., 2017).

Nanopore sequencing offers potential benefits in molecular characterization of GM products compared with PacBio, since it delivers raw data in real-time, is relatively easy to manipulate, and has low setup costs. The MinION from ONT is a portable device that has been successfully assessed for detection of unauthorized GM products (Fraiture et al., 2018). Further assessment demonstrated the capability of the MinION to determine the full molecular characterization of three transgenic crops (ryegrass, canola and clover) within 48 hours.

Although, new guidelines are emerging from regulatory bodies to generate pre-market submission of data using whole genome sequencing (Health Canada, 2019) and NGS (UCD Centre for Food Safety et al., 2018). Sequencing approaches are typically being used in tandem, for example Sanger sequencing with SGS or SGS with TGS, for verification and validation purposes during risk assessment of GM crops (Boutigny et al., 2019).

In short, GM forage species including Alfalfa (Barros et al., 2019), Switchgrass (Dumitrache et al., 2017), Sorghum (Rooney et al., 2007) have been characterized adopting the same technologies used for GM food. However, the expression of introduced traits in plant parts not used for food, should be considered. For instance, a GM plant can be produced with specific production of the Bt toxin only in leaves, preventing insect attack and removing the exposure of humans to the compound when consuming its grains (OECD, 2003). However, in such cases those plant parts would substantially increase the exposure of animals to the GM toxin when consuming them, and this difference must be taken into account in the molecular characterization.

### GM Traceability

GM traceability describes a system that enables tracking of GM food/feed products at all stages of the supply chain (Giraldo et al., 2019). Detection methods for GM products in different matrixes or substrates, such as grain, flour and forage, are not only important to ensure legality and traceability, but also to comply with GM labeling regulations (European Parliament, 2003).

Methods for GM detection and identification usually rely on certified reference materials that are in powdered form, however, routine detection must be performed in different agricultural and food products (Cankar et al., 2006). The selection of DNA extraction protocols is of crucial importance, since the DNA can be present in low amounts, carrying inhibitors or degraded (SanJuan-Badillo et al., 2014). Therefore, the extraction method should be evaluated for each agricultural product, guaranteeing high DNA yield and purity (Turkec et al., 2015).

The method chosen to comply to traceability and labeling requirements, should be sensitive enough to detect the transgene(s) at levels below the corresponding jurisdiction tolerance threshold (e.g., 5% in US, 1% AU, and 0.9% in EU) (Ramessar et al., 2010). Additionally, it should be able to detect the transgene(s) from raw agricultural commodities entering the feed production chain. For instance, fresh leaves, dry leaves (hay), pollen, seeds, tillers or stems, and forage that could enter the feed chain as unprocessed material (Ardizzone et al., 2018; Giraldo et al., 2019).

Currently, qPCR is the standard method used in national reference laboratories for detection and quantification of GM events (Dalmira et al., 2016). The requirement for reference material to be used as calibrants, which sometimes are not commercially available, limits its effectiveness (Dobnik et al., 2016; Dalmira et al., 2016). The GM product detection process followed by national reference laboratories consist of two consecutive steps; first, a qPCR screening of vectors commonly found in GM products, such as the 35S promoter from cauliflower mosaic virus, *Agrobacterium tumefaciens* (tNOS) and selectable markers. Then, the samples with a potential presence of GM materials, are tested using the corresponding GM event-specific method (Fraiture et al., 2017).

Droplet digital PCR (ddPCR) technologies use the same DNA amplification principles as qPCR, but the technologies can provide a higher quantification precision through partitioning PCR mix into thousands of nanoliter-sized droplets in which PCR amplification is carried out (Dobnik et al., 2016; Dalmira et al., 2016). Features such as absolute quantification, avoidance of using standard curves, and high resilience to inhibitors, makes ddPCR a promising alternative for GM event detection (Rački et al., 2014; Corbisier et al., 2015).

SGS technologies have also been proposed to comply with the requirements for GM traceability due to the ability to detect all target sequences in multiple samples without the development and validation of target-specific methods and reference material (Arulandhu et al., 2018). However, the requirement of bioinformatics knowledge for data analysis and more sophisticated devices limits its use in routine GM event detection (Park et al., 2017). **Figure S4** compares qPCR, ddPCR, and SGS in terms of GM identification and quantification, multiplexing capacity and ability to detect known and unknown sequences. It also underlines that the cost and processing time increase from qPCR to ddPCR and to SGS.

In recent years, significant effort has been performed to replace the time-consuming and expensive qPCR screening procedure (Holst-Jensen et al., 2016; Salisu et al., 2017). As a result, other technologies are been evaluated including ddPCR (Dalmira et al., 2016; Dobnik et al., 2015; Köppel et al., 2015; Demeke et al., 2016; Dobnik et al., 2016; Gerdes et al., 2016; Głowacka et al., 2016; Iwobi et al., 2016; Grelewska-Nowotko et al., 2018; Niu et al., 2018; Corbisier and Emons, 2019; Giraldo et al., 2019), SGS (Willems et al., 2016; Fraiture et al., 2017; Arulandhu et al., 2018), DNA enrichment approaches (Arulandhu et al., 2016) and combined strategies of DNA walking and SGS (Fraiture et al., 2017).

However, all the above methods are lab-based and time consuming, so that the development of rapid and portable devices with more routine throughput screening methods are likely to continue into the immediate future. Recent research related to *on-site* detection of GM crops include; loop-mediated isothermal amplification (LAMP) (Hardinge et al., 2018; Singh et al., 2019; Loo et al., 2019), LAMP and a lateral flow biosensor (Cheng et al., 2017), PCR and a lateral flow biosensor (Gao et al., 2019), handheld field-portable qPCR systems (Nguyen et al., 2018; Russell et al., 2018), and nanopore sequencing (Fraiture et al., 2018; Russell et al., 2018).

Technologies to fulfil regulatory requirements for GM food traceability have been established. The qPCR-based method is suitable for a high-throughput screening of transgene(s), and the ddPCR-based approach can provide a higher accuracy of the measured GM product concentration, especially at a low level. Although, the same approaches of GM food may be used for GM feed to comply the regulatory requirements, for GM feedstuff special considerations should be given to the use of plant parts not used for human food and by-products from other industries using GM plants (OECD, 2003).

### Environmental Safety Studies

Environmental risk assessments (ERA) aim to determine whether a new GM crop variety has direct effects on the natural environment (Hilbeck et al., 2011; Devos et al., 2016). Although a range of factors, such as effects on biodiversity, modification of soil and water quality, and disease and weed control, must be considered in this process, the major concern of ERA is gene flow (GF) of the transgene(s) to wild relatives (Warwick et al., 2009).

GF is a result of the movement of gametes or individuals from a specific population to another, which may generate a significant change in the allele frequency of the receiving population (Slatkin, 1987). This phenomenon not only has been observed between populations of the same species, but also between closely related species (Wilson and Manhart, 1993; Bartsch et al., 2002). In case of natural plant populations, such movement can happen *via* seeds, vegetative propagules or pollen and its importance varies among plant species (Tsatsakis et al, 2017). General approaches to quantify GF use foreign herbicide and antibiotic resistance genes to provide insight into the rates and importance of hybridization (Mallory-Smith et al., 2015). However, morphological and molecular markers are also required to assist with rapid identification or to identify/confirm hybrids.

To allow GM and non-GM crops to exits in mutual tolerance and minimize undesired GF, a concept defined by the term “coexistence” was introduced. Coexistence refers to the right that consumers have to choose between conventional, organic, and GM crop production, in compliance with the legal obligations for labeling defined in each jurisdiction (Devos et al., 2009). Although, different varieties of the same species have coexisted prior to appearance of GM crop variety, the need for the strategies to manage the consequence of inadvertent fertilization have become more outstanding with the commercial release of GM crops (Ramessar et al., 2010).

A potential risk of transgenes GF is cross-pollination between GM crops and native species, which has been intensively discussed since the commercial realize of GM plants (Messeguer, 2003; Kuparinen et al., 2007; Warwick et al., 2009; Tsatsakis et al., 2017). Pollen-mediated GF has been reported during production of commercial GM crops including maize, rapeseed, cotton, soybean, and creeping bentgrass (Reichman et al., 2006; Chifflet et al., 2011; Baltazar et al., 2015; Rizov and Rodriguez-Cerezo, 2015; Loureiro et al., 2016; respectively).

One of the most common coexistence measures to reduce pollen-mediated GF is isolation distances. It is defined as the minimum distance between GM and non-GM crop fields of the same species that should prevent a cross-pollination rate from reaching to threshold levels (Cunliffe et al., 2004). Multiple factors, such as population size, distance, and flowering synchrony between donor and receiver fields, as well as local wind conditions, all influence the determination of an appropriate isolation distances (Devos et al., 2009).

Different isolations distances have been determinate to avoid or minimize cross-fertilization between GM and non-GM fields. **Table 1** describes different isolation distances required for the four major GM crops (maize, canola, soybean, and cotton), to maintain cross-fertilization ratio below legal tolerance thresholds. For instance, to maintain cross-fertilization levels below 1% an isolation distance of 20 m is require for maize (Baltazar et al., 2015), 9 m or more for cotton (Baltazar et al., 2015), and 5 m for soybean (Rizov and Rodriguez-Cerezo, 2015).

**Table 1:** Cross-fertilization level in different isolation distances for the major forage crops commercially available.

Crop	Isolation distance	Cross-fertilization level	Reference
Maize	50 m	<0.5%	Sanvido et al., 2008
	20 m	<1%	Baltazar et al., 2015
Canola	30 m	<0.03%	Staniland et al., 2000
	33–200 m	<0.015%	Cai et al., 2008
Soybean	5 m	0.9%	Rizov and Rodriguez-Cerezo, 2015
	10 m	0.1%	
Cotton	10 m	<0.9%	Loureiro et al., 2016
	>9 m	<0.1%	van Deynze et al., 2005
Alfalfa	150 m	1.39%	Fitzpatrick et al., 2003
	500 m	0.08%	

In GM forage species, undesired pollination can be the major type of GF, since forage grass species typically exhibit a highly outcrossed nature and are wind-pollinated (Holme et al., 2013). Smith and Spangenberg (2016) reviewed the most important coexistence strategies in outcrossing forage species, highlighting that a coexistence framework for the dominant cross-pollinated grain crops (canola and maize) is already well established in Europe, as well as alfalfa in the US. Hence, existing principles such as development of detection techniques, segregation and agronomic management can be applied to other forage crops when developing coexistence frameworks.

Compared with commercially available grain crops, studies on pollen-mediated GF in wind-pollinated forages is scarce. A study on transgenic alfalfa (*Medicago sativa* L.) using the *cp4 epsps* transgene as marker, showed that a 500-m isolation distance is required for maintenance of the cross-fertilization level below 0.1% (Fitzpatrick et al., 2003). Rigid ryegrass (*Lolium rigidum*) gene flow was reduced from 37.8% at 0 m and 0.93% at 100 m (Busi et al., 2008). In other species with high levels of self-compatibility such as barnyardgrass (*Echinochloa crus-galli*), cross-fertilization rates at 0-m distance was 12.5% (Bagavathiannan and Norsworthy, 2014) and for tall fescue (Festuca arundinacea) 5% were detected at 50 m and less than 1% at 150 m (Wang et al., 2004).

Transgenic GF of the commercial GM grains has been intensively studied and discussed as a main part of ERA, some of such information can be apply to GM forage crops, the potential risks may be depending on the nature of transgenic traits. For GM forages considerations such as life cycle, since forage crops are largely perennial not annual or biannual, can have repercussions on pollen flow and it can remove management of gene flow through farming systems temporally.

Different regulations and laws about GM plants create complexities for the movement of agricultural products between borders and has a significant impact on international trade. The Cartagena Protocol on Biosafety is an international treaty that aims to protect biodiversity from the potential risk of GMO resulting from modern biotechnology. Such Protocol dictates that GM plants should follow the precautionary principle, which states that “In order to protect the environment, the precautionary approach shall be widely applied by States according to their capabilities” (Secretariat of the Convention on Biological Diversity, 2000). Therefore, standardized methods of ERA for GM crops has not been established and it is managed by individual laws in each country. Usually, a case-by-case assessment needs to be performed and the methods used for GM food and feed are the same.

### Feed Safety Studies

The vast majority of forage crop products are fed to livestock; hence, any human consumption of GM feedstuff is an indirect effect and can easily be regulated and mitigated to ensure complete safety. Feed safety studies examine whether the genetic modification could unintentionally increase the potential toxicity or allergenicity of the transgenic plant for humans or animals, as well as changes in nutritional characteristics (Pauwels et al., 2015). As forage crop products are predominantly eaten by animals and human consumption of GM feedstuff represents an indirect effect, and this type of assessment should be unique to GM feed. Feeding studies focus on answering three main issues; substantial equivalence, the safety of the new crop for humans and animals, and the safety of the product derived from animals raised on transgenic feed. Those issues are comprehensively discussed in Aumaitre et al. (2002) and Ramessar et al. (2007).

According to The Organization for Economic Co-operation and Development (OECD), the substantial equivalence concept refers to the idea that existing food products can serve as a basis for comparison when assessing the nutritional value and safety of food modified by modern biotechnological methods (OECD, 1993). This comparison helps quantify the effect of the transgene as well as understanding the variation in the natural species for the trait being modified. The analysis of chemical composition would serve as the first step for the nutritional evaluation. Such comparison is, however, largely based on the premise that an existing cultivar with a history of safe use, can serve as a comparator when evaluating the safety of a GM food/feed (Flachowsky et al., 2012). The type of comparators, key characteristics selected, and interpretation of compositional data may vary change among different countries. The OECD, an intergovernmental organization in which representatives of 30 industrialized countries in North America, Europe, and the Pacific, have consensus documents about common GM plants, which are a valuable source to ensure a consistent assessment (OECD, 2000).

Some examples of GM products that passed the substantial equivalence test include a maize line with the sb401 gene incorporated to increase lysine content, the equivalent test with a conventional protein quality maize (Nongda 108) showed that the lysine-rich maize was safe (Tang et al., 2013). The compositional equivalence of a herbicide-tolerant rice (Bar 68-1) exhibited no significant differences, when compared with its isoline (D68) (Li et al., 2008). The evaluation of a *Bt* soybean (products Mon 87701 * Mon 89,788) that confers pest resistance and glyphosate resistance, concluded that *Bt* soybean was as safe as its traditional counterparts (Berman et al., 2009). Similarly, Bollgard II cotton (event 15985) with insect resistance properties was compared with traditional cotton varieties, and the results demonstrate that it was as safe and nutritious as conventional cotton for food and feed use (Hamilton et al., 2004).

When substantial equivalence of GM feed product to its traditional analogues cannot be concluded, a further assessment comprises of the following steps: *in silico*, or *in vitro* preliminary studies, the study of nutritional value of the products; quotas in the diets of animals; methods of use in nutrition, and during lactation; digestibility, evaluation of intake of individual components (if the expected intake is more than 15% of the daily requirement); impact on the intestinal microflora (if GM product contains live microorganisms) (Levitsky 2016). The safety assessment of each country can be different and in some jurisdiction animal experiments are not required. For instance, the OECD consider that GM plants where compositional analyses demonstrate no significant differences from the comparator, animal feeding studies with target species will add little to a safety assessment, so that nutritional equivalence can be assumed (OECD, 2003).

As a part of assessment for animal safety issues, the *in silico*, or *in vitro* studies also can provide an estimation of the impact of GM feed in the nutrition of target animals, prior to the feeding studies involving animals. *In silico* evaluation may serve to identify changes in key nutritional components and *in vitro* simulated gastric and intestinal fluids to study the digestive stability of novel proteins (ILSI, 2007; DBT, 2008). All laboratory studies should be conducted according to the internationally recognized guidelines (OECD, 1998). The information obtained from these studies help to determine the need for future *in vivo* studies with target animals (EFSA, 2008). The OECD has developed guidelines for different animal and *in vitro* testing (OECD, 1995: Guidelines 401, 407, 408, 414, 415, 416, 420, 421, 423, 425, 451, 452, 453, 474, 475, 478, 486).

Tudisco and Infascelli (2014) used an *in vitro* gas production technique to compare the nutritional value of GM corn and soybean with their traditional counterparts. They found that the fermentation kinetics of GM corn and gas production of GM soybean were respectively faster and lower compared with their counterparts. Another *in vitro* experiment using batch fermentation models assessed the possibility of transfer of cry1Ab transgene to porcine jejunal microbiota, concluding that the transfer of transgene was not detected (Buzoianu et al., 2012a).

Swiatkiewicz et al. (2014), comprehensively reviewed the published information on health status, blood parameters, immunological characteristics, histopathological examination of cattle, poultry and fish. Concluding that the quality and potential risks for human consumption of livestock products such as meat, milk, and eggs and the metabolic parameters were not significantly affected when livestock were fed with commercialized GM crops. Although, there is a large proportion of studies on livestock animals, most of them focuses on the possibility of horizontal gene transfer (HGT) from GM crops to animal tissues.

Feeding test, using model animals has been conducted for a safety assessment of GM food crops. The main animal species, which would consume the GM feed products are, however, livestock, such as cattle, sheep, swine, and poultry (Nadal et al., 2018). Cattle (dairy and beef) and sheep belong to the ruminant category, with a unique digestive system that gives them the ability to obtain nutrients from forage crops by breaking down its cellulose content in a special stomach compartment using microbial actions. On the other hand, swine have a monogastric digestive system with an enzymatic stomach very similar to humans, and poultry have an avian digestive system without teeth to chew the feed (France et al., 2006). Cattle and sheep are herbivores, whereby their diet is mainly forage-based, while swine, poultry, and humans are omnivores, so they eat mostly grains and some plants (Flanders and Gillespie, 2015).

Until now the safety assessment of GM plants has focused in human exposure, animal safety while not ignored, has received less importance (Aumaitre et al., 2002). Pigs have been widely used as models for humans, because of their similar gut anatomy and physiology (especially mucosal immunity) and nutritional requirements (Ladics et al., 2010). Rodent tests have received the greatest importance from regulatory bodies, especially for toxicological and allergenicity studies of the products of introduced genes. However, these animals are not usually feed with the entire GM plant or their by-products, while livestock animals have greater exposure to GM feedstuff, compromising a high percentage of their diet on a daily basis and often for their complete lifespan (Aumaitre et al., 2002). Additionally, variations in the livestock system can determine the evaluation profile, for instance, forage grasses will be relevant to ruminant species and have little to no value to monogastric species.

Due to their anatomical and eating habits differences, it is impossible to assess the safety of a transgenic GM feed with a single and unified test. A more specialized case-by-case approach is essential for the safety assessment of GM feed. Development of standardized *in silico* and *in vitro* approaches, can reduce time and cost for this process and avoid the use of animals. When required, *in vivo* feed studies offer the possibility of conducting feeding trials in the target species, something not possible with GM food in humans.

### Toxicological Studies

The purpose of toxicological studies is to characterize intended changes and detect active substances or compounds that could have unexpected toxic effects for non-targeted organisms (Van Haver et al., 2003). All toxicity assessment for GM material should be performed based on a case-by-case approach, considering the toxicological profile of new introduced substances (Domingo, 2007).

The methods to assess the toxicity of a specific compound in the body, usually compromise the use of animal studies, considering the target species and the critical effects (Levitsky, 2016). However, new strategies to identify GM feed anti-nutrient or toxicants include research on the in-planta metabolism pathway, such as “-omics” techniques that may generate a better understanding of the complex pleotropic effects of new plant cultivars (Fernandez and Paoletti, 2018). Additionally, *in vitro* assays with gastric enzymes, cultured cell lines, receptor proteins, and *in vivo* animal studies can be performed (Van Haver et al., 2003).

High-throughput “-omics” profiling techniques, which involve the use of metabolomics, transcriptomics, and proteomics, have been suggested as a nontargeted approach to detect unintended effects in GM crops (Ricroch, 2013). Profiling studies using omics techniques include GM glyphosate-tolerant soybean, where some specific metabolites were different compared with the isogenic line and the results were explained by modifications in the regulation of the shikimate pathway (Garcia-Villalba et al., 2008). Nevertheless, a GM stacked rice carrying the herbicide-resistant gene *bar* and insect-resistance *cry*, was found substantially equivalent to its conventional genetic breeding and natural genetic cultivars, when their proteome profiles were compared (Gong et al., 2012). A review of the safety assessment of GM crops using omics techniques, indicated that transgenesis has less unintended impacts than conventional breeding (Ricroch, 2013). Another study showed that there were more transcriptomic alterations in mutagenized plants than transgenic plants (Batista et al., 2008).

Several toxicological studies in GM feed using omics techniques, involve the analysis of fungus or their secondary metabolites. For instance, mycotoxins, which are undesired substances produced by crop-related fungus. In hybrids *Bt* maize, one of the principal components of feeding formulas for livestock, plants experienced less fumonisin concentration compare with its isoline (Bowers et al., 2014). It was hypothesized that the reduction of fumonisins was due to the pest reduction in the GM maize, since the fungus spore migration and colonization may be facilitated with damages from insects. Therefore, it could be concluded that GM corn can provide reductions in the risk of fumonisins contamination, but not increment of toxicological risk for animals.

Similarly, in the transgenic high-energy perennial ryegrass, an evaluation of alkaloids, secondary metabolites produced by endophytic fungus, found that the alkaloids concentration in transgenic plants was same or lower compare with the isogenic line. The lower alkaloid concentration could be partly attributed to higher growth of transgenic plants, which could generate a dilution effect in the modulation of fungal biomass (Giraldo et al., 2018).

When performing *in vivo* studies, toxicology acute (14 days studies), subacute (28 days studies), chronic (90 days studies), or specific toxicity (reproductive, mutagenicity, etc.) assessment can be considered (Levitsky, 2016). In a chronic study feeding mice with crushed *Bt* cotton seeds, *cry* genes and *tnos* promoter were detected only in intestinal tissue, while they were not found in stomach, blood, liver, kidney, heart, and brain (Sajjad et al., 2014). In long term studies (>100 days), no toxic effects were found in cattle and chickens fed with *Bt* maize. They concluded that short fragments of plant chloroplasts (<200 base pairs) can be detected in blood lymphocytes of cattle, but DNA fragments were not detected in other organs investigated (muscle, liver, spleen and kidney) (Einspanier et al., 2001). Similarly, some small changes in the metabolic profile of sheep fed with *Bt*176-maize were found when compared with non-GE maize, according to the authors such changes did not represent a health hazard (Snell et al., 2012). In pigs fed with *Bt* maize (MON810 event), although all serum biochemistry parameters were within the normal reference interval for pigs, small differences were reported. The authors concluded that the differences were the result of a lower enzyme-resistant starch in the GM compare with the non-GM control (Buzoianu et al., 2012b).

Safety assessments of GM feeds should consider the maximum level present in any plant part consumed by animals or in any by-product used as a feed ingredient, since the introduced traits can express differently in the plant parts, affecting the concentration of novel proteins. This can have implications in the level of exposure, selection of comparators and determination of the novel protein concentration used in acute/sub-chronic toxicity studies (OECD, 2003).

In short, a case-by-case approach is also required for toxicological assessment of GM crops, and the assessment procedure has not been standardized. A comparison with conventional counterparts has been a common approach for GM food products, and a similar approach may be used for forage products. A more cautious and stringent examination may be required for GM feed, due to that a wider range of plant organs may be used for animal consumption than those for human consumption, and storage conditions of the GM forage products may be less uniformed and controlled than GM food.

### Allergenicity Studies

Allergenicity and toxicological studies may be assessed at once, since both are designed to detect newly expressed substances. Allergenic reactions can cause more severe symptoms, but usually to only some individuals, while toxicity is predictable and reproducible between individuals as it affects the majority of exposed individuals with only minor differences in susceptibility (De Santis et al., 2018).

In US, concerns about potential risks for allergy have arisen from GM food crops, including one under development for several times. A 2S albumins gene from Brazil nut was introduced into a soybean cultivar, for a purpose of nutritional enhancement. The transgene products, however, were identified to have potential allergic risks for human, especially those with allergy to the Brazil nut, and development of the GM soybean cultivar was suspended (Moreno and Clemente, 2008; Delaney, 2015). Concerns for the Cry9C protein, a type of insect pest resistance protein from *bacillus* also arose, due to a higher stability to heat and possible prolong time for digestion (Wiedinmyer et al., 2000). As a consequence, the StarLink maize, an unauthorized maize containing the Cry9C transgene, was not approved for human consumption by the US authority (Zhang et al., 2016).

In case of a GM feed safety assessment, both human and animals may need to be included in an allergenicity study as test subjects. An allergenicity assessment for animals could be performed with a similar approach to that for human. There is, however, currently no standardized procedure to predict allergenic reactions to non-endogenous proteins even in humans. The European Food Safety Authority (EFSA) has recommended using animal models to evaluate the sensitizing potential of novel proteins on a case-by-case basis (Marsteller et al., 2015). The most common species to assess GM allergenicity are rodents, also referred to as a rat 90-day evaluation, which is now compulsory in the EU for new GM crops (Hong et al., 2017). However, the published studies on rats, mice, and pigs, aimed to assess the allergic risk of humans (Ladics et al., 2010), using animals mostly as food allergy models.

The most common approaches to assess allergenicity include amino-acid sequence homology, *in vitro* digestibility tests, serum screening and animal models (Van Haver et al., 2003). Amino acid sequence homology or similarity uses bioinformatic methods to determine the possibility, that a novel protein can be closely similar to a known allergen that can create a risk of cross-reactions (Naegeli et al., 2017). However, such bioinformatic methods cannot predict the likelihood that the novel protein might become a *de novo* allergen, so other methods like *in vitro* digestibility tests, serum screening, and animal models may need to be used (Ladics et al., 2010). The *in vitro* pepsin resistance assay is the most commonly used protein digestion test, which provide information about the susceptibility of a novel protein to digestion. This assay can be used as an additional evidence of possible adverse reactions to GM food/feed, since gastrointestinal digestion can affect the immunogenicity of dietary proteins related to both IgE and non-IgE reactions (Naegeli et al., 2017).

Serum screening and immunoassays are alternative ways to assess endogenous allergens, using sera from individuals with relevant allergies. Although, these types of assays are the current reference method for *in vitro* detection and definition of an allergenic proteins, detection of allergic animals pose limitations for the applicability in the safety assessment of GM feed. Alternatively, there are other analytical and molecular profiling techniques, which can serve as alternative tests for the comparative assessment of the endogenous allergenicity between the GM plant and its non-GM comparator (Fernandez et al., 2013).

Information on immunological responses, and particularly allergenic reactions on livestock fed with GM products is scarce and is likely to need more extensive research. Nevertheless, allergenicity assessment of GM feed offers the advantage of direct evaluations in target species, something not possible with GM food in humans.

### Horizontal Gene Transfer

HGT refers to the movement of a genetic material to a living cell or organism across boundaries between species. In the case of GM organisms, movement of transgene(s) into other species, especially microorganism, or natural population of the taxonomically related species have been concerned, as such transfer may have impacts on human/animal health, and natural environments. (EFSA, 2007; EFSA, 2009; ADAS, 2013; Nicolia et al., 2014; Van Eenennaam and Young, 2014; Giacomo et al., 2016). This section relates to environmental, feed safety, toxicological, and allergenicity studies, since the transfer of recombinant DNA into other organisms can affect the health of humans, animals, and the environment.

Although assessment of the digestion fate of recombinant DNA or its new proteins can be performed using *in vitro* gastric or intestinal fluid-based digestion systems to assess HGT in bacteria (Fuchs et al., 1993; Wehrman et al., 1996; Entransfood 2004; Sharma et al., 2004; Bertrand et al., 2005), studies using cattle species have been also commonly conducted evaluating the digestive process of recombinant DNA and proteins from feedstuff (Faust and Miller, 1997; Ash et al., 2000; Aulrich et al., 2001; Jennings et al., 2003; Aumaitre, 2004; Phipps et al., 2005; FASS, 2006; Rizzi et al., 2012; Swiatkiewicz et al., 2014; Van Eenennaam and Young, 2014; Levitsky, 2016; Van Eenennaam and Young, 2017; Nadal et al., 2018). For example, recombinant DNA has been found in ruminal solid phase and duodenal digesta of cattle, but the DNA was not detected in liquid ruminal and duodenal phase, as well as milk, blood, and faeces. Those results indicate a rapid degradation of the transgene in the first digestive stages (Phipps et al., 2003).

In studies using cows fed with transgenic maize, recombinant DNA was not detected from milk (Faust and Miller, 1997; Giacomo et al., 2016), blood, muscle, kidneys, liver, or spleen (Einspanier et al., 2001). Also, recombinant DNA was not detected from milk of cows grown with feed including up to 26% of transgenic glyphosate-tolerant soybean (Phipps et al., 2002). Similarly, in studies on poultry fed with transgenic maize, recombinant DNA was not detected from muscle, kidneys, liver, and spleen, as well as eggs (Einspanier et al., 2001). Only a little possibility of incorporation of recombinant DNA into the genomes of human or animal of digestive organs has been suggested from some of these studies, and the majority of the studies concluded that such risk of horizontal transfer of transgene is insignificant (Chambers et al., 2002; Ramessar, et al., 2007; Levitsky, 2016). As the risk of incorporation of recombinant DNA into germ cells should be even lower, the possibility of inheritance of the recombinant DNA into the following generation should be insignificant.

In case of GM feed products, possible risks on human health may need to be also considered, as the products are indirectly consumed by human *via* cattle. The rapid digestion process of recombinant DNA, evidenced by the studies described above, suggests that the indirect risks on human health are low. The safety of food products produced from animals feed on transgenic crops has also been widely studied, and in the majority of studies, any recombinant DNA was not found in animal products. However, in a couple cases, short fragments of the recombinant DNA were detected in milk (Phipps et al., 2002; Agodi et al., 2006). The authors, however, interpreted their presence as a contamination of faecal or airborne material with feed particles (Agodi et al., 2006).

Most of the scientific finding till the date has not found significant risk directly related with the consumption of GM crops and these findings can be extrapolated to forage species. In general, proteins derived from recombinant DNA, as any protein, are degraded in the gastro-intestinal tract, while dietary DNA is not totally degraded and, in some cases, small fragments can be found into animal tissues (Nadal et al., 2018).

## Conclusion

The use of molecular breeding technologies such as genetic modification and genome editing in forage crop species can help farmers address the challenges of climate change, sustainability, and global food security. Information about the safety assessment of GM forage crops intended only for animal feeding is scarce, even though most of GM products and its biomass is destined for livestock animals feeding. The regulatory assessment scheme is designed for GM food and a similar approach can be used for the assessment of forage crops considering the differences in risk profile of the contrasting outcomes.

The same techniques used for molecular characterization, GM traceability, environmental safety studies and HGT can be used for both GM food and feed. However, specific adjustments to the techniques may be required, considering that parts of the GM plant used to feed livestock may have different concentrations of the novel proteins, changing its level of exposure. Feed, toxicological, and allergenicity studies for GM feed only destined for animal consumption are not well defined. The design of specific strategies to cover GM feed safety can be more targeted for the safety of species that are going to consume the crop, while also potentially having a lower regulatory cost.

A new framework for the risk assessment procedure, for both GM food and feed, is necessary in order to make a more efficient use of resources and avoid unnecessary evaluation. The final aim should be to assess GM novel crops in a more effective way, to increase the commercialization of products with potential to provide economic and health benefits to consumers and producers.

## Author Contributions

PG has written the manuscript under the supervision and drafting of HS, NC, and KS. The review was finally edited by HS, GS, NC, and KS.

## Funding

This work was supported by funding from Agriculture Victoria Research and an Australian Government Research Training Program Scholarship for PG.

## Conflict of Interest

The authors declare that the research was conducted in the absence of any commercial or financial relationships that could be construed as a potential conflict of interest.

## Supplementary Material

Figure S1 Difference between GM food and feed.

Figure S2 Different breeding techniques and its requirement for safety assessment before commercialisation.

Figure S3 Pre and pos-marketing issues to be solve before GM crops commercialisation.

Figure S4 Comparison of qPCR, ddPCR and NGS.
