# Supplementary material for: Safety Assessment of Genetically Modified Feed: Is There Any Difference From Food?
Source: Front Plant Sci. 2019 Dec 11;10:1592. doi: 10.3389/fpls.2019.01592 (PMC6918800; doi:10.3389/fpls.2019.01592)

Supplementary material

Figure S1: Difference between GM food and feed.

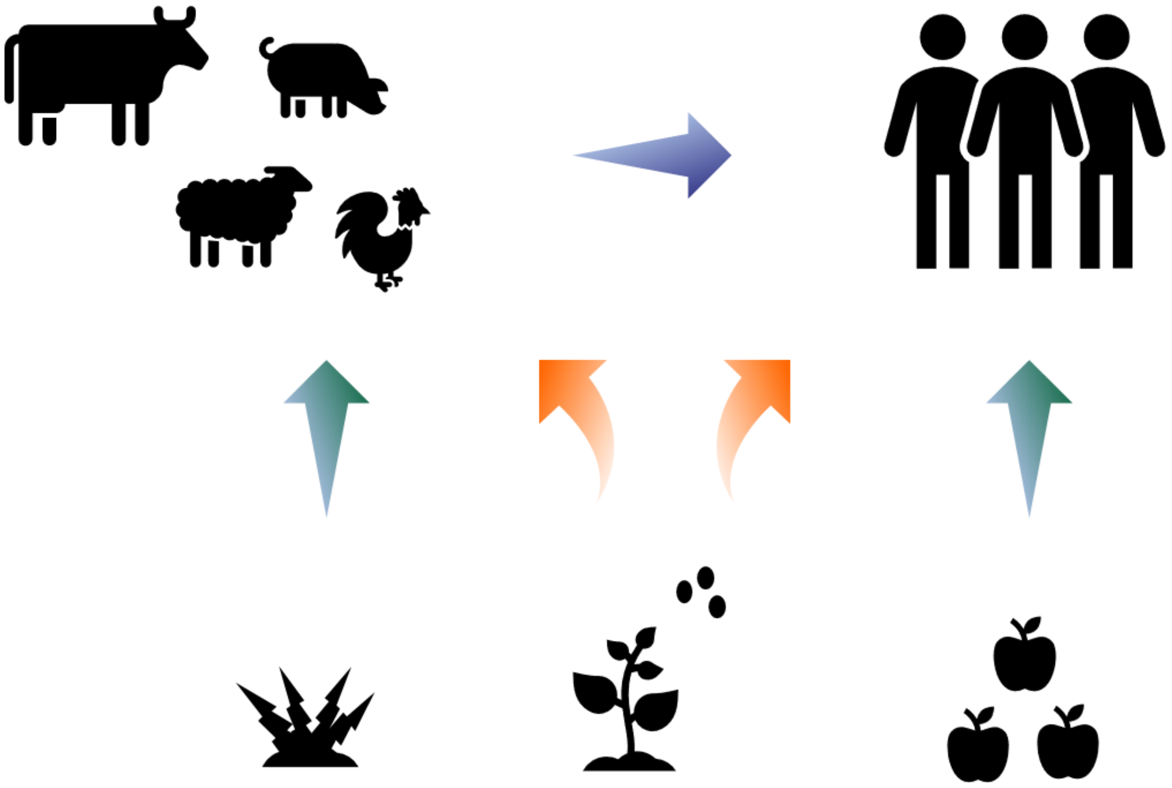

Figure S2: Different breeding techniques and its requirement for safety assessment before commercialisation.

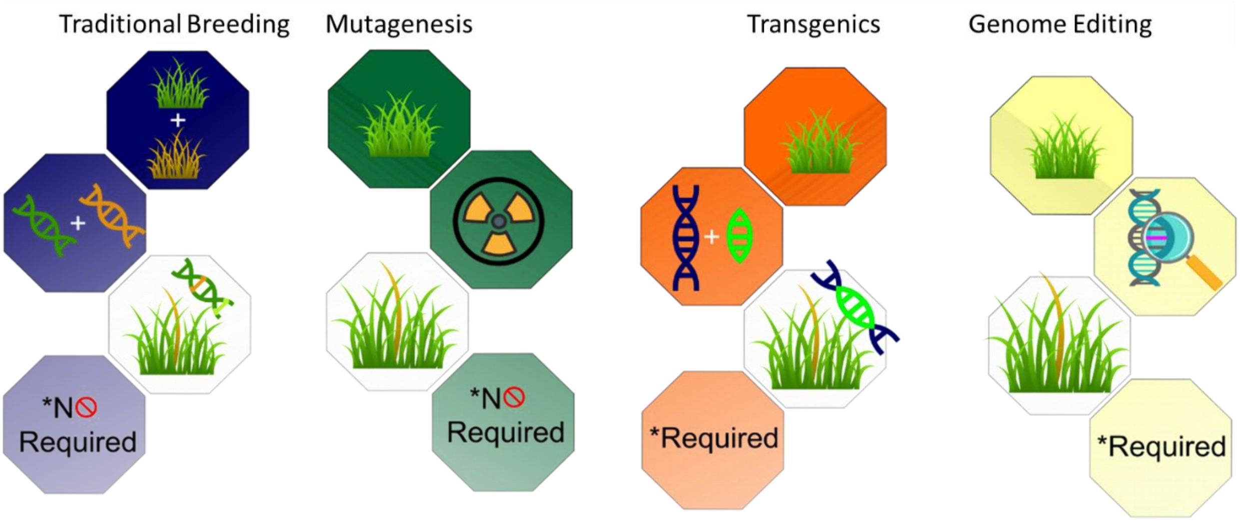

Figure S3: Pre and pos-marketing issues to be solve before GM crops commercialisation.

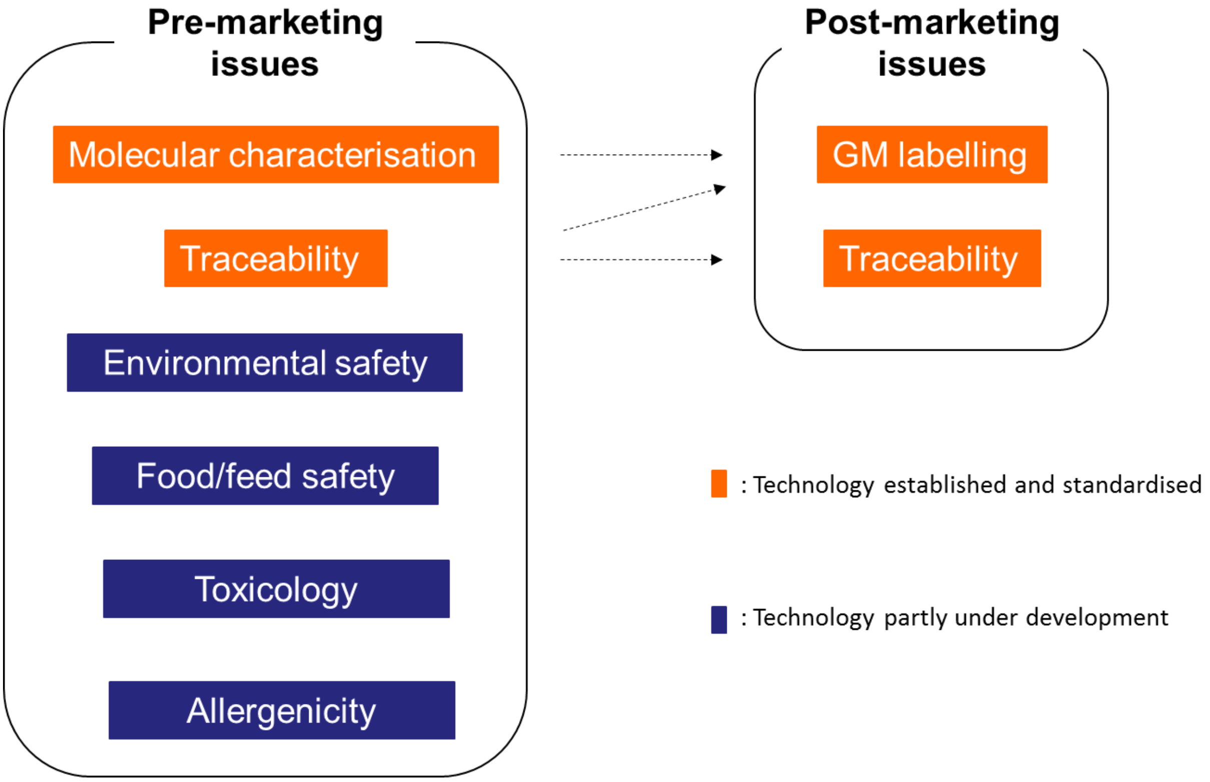

Figure S4: Comparison of qPCR, ddPCR and NGS.

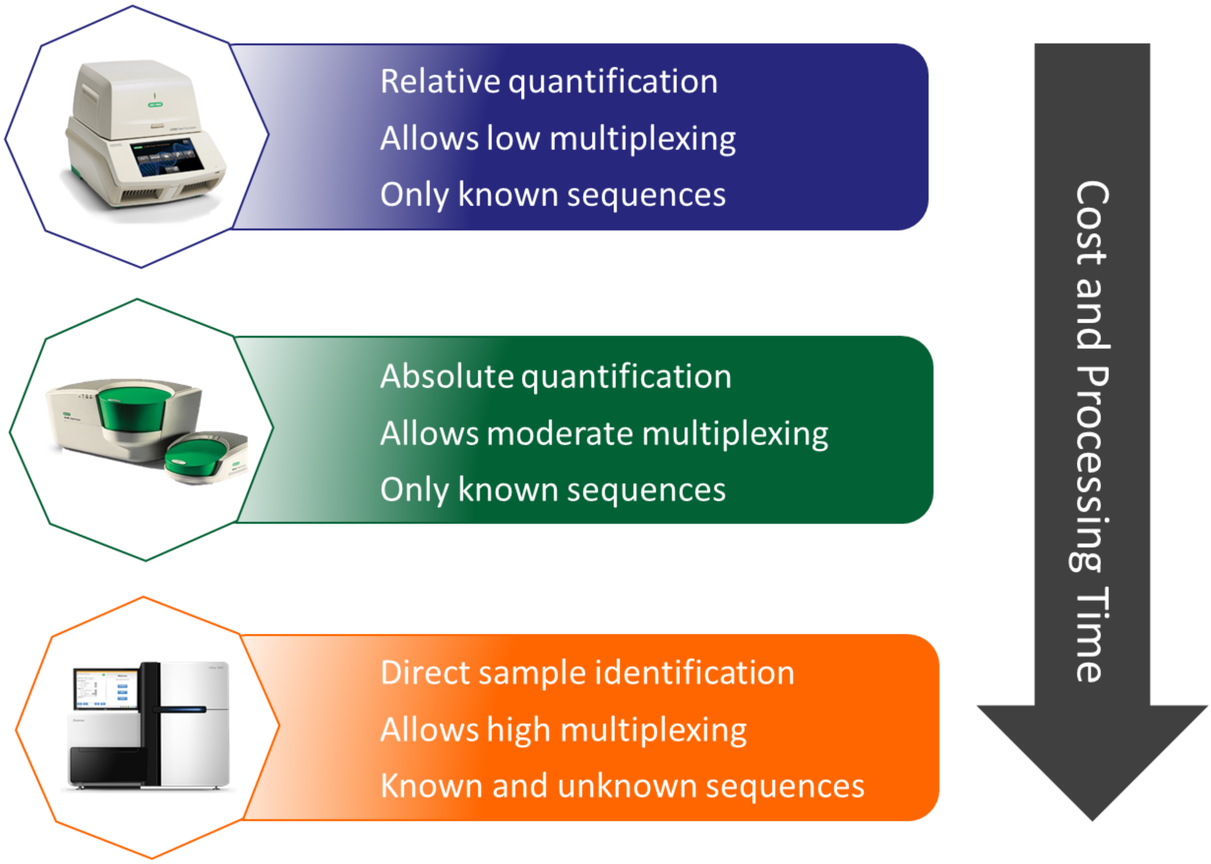

Supplement: Figure S1 — Difference between GM food and feed. [file Presentation_1.pdf]
